# Optical coherence tomography surpasses fundus imaging and intracranial pressure measurement in monitoring idiopathic intracranial hypertension

**DOI:** 10.1038/s41598-025-96831-9

**Published:** 2025-04-28

**Authors:** Yumin Huang-Link, Sanna Eriksson, Jan Schmiauke, Ursula Schmiauke, Mats Fredrikson, Max Borgström, Ge Yang

**Affiliations:** 1 Division of Neurology, Department of Biomedical and Clinical Sciences, Linköping University, Linköping, Sweden; 2 Faculty of Medicine and Healthy Sciences, Linköping University, Linköping, Sweden; 3 Forum Östergötland and Department of Biomedical and Clinical Sciences, Linköping University Hospital, Linköping, Sweden; 4 Division of Psychiatry, Department of Biomedical and Clinical Sciences, Linköping University, Linköping, Sweden; 5 Huizhou Aier Eye Hospital, Huizhou, China

**Keywords:** Fundus imaging, Idiopathic intracranial hypertension, Intracranial pressure, Optical coherence tomography, Papilledema, Retinal nerve fiber layer, Visual system, Neuroscience, Neurology

## Abstract

We aim to evaluate the retinal nerve fiber layer (RNFL) thickness measured with optical coherence tomography (OCT) in comparison with papilledema grade, and to assess the relationship between RNFL thickness, papilledema grade, and intracranial pressure (ICP) in idiopathic intracranial hypertension (IIH). Sixty-five patients with active IIH (AIIH) with papilledema, 39 with chronic IIH (CIIH) without papilledema and 80 healthy controls (HC) were examined with OCT and fundus imaging. RNFL thickness, papilledema grade and ICP level were assessed in 55 with AIIH and 26 with CIIH. RNFL thickness was significantly higher in AIIH compared to CIIH or HC. RNFL thickness correlated strongly with papilledema grade (coefficient 0.78, p < 0.01) and moderately with ICP (coefficient 0.569, p < 0.01). RNFL thickness was associated with papilledema progression (R^2^ = 0.656, p < 0.01): specifically, with increases of 9 µm from normal to mild grade (p > 0.05), 91 µm from normal to moderate (p < 0.01), and 214 µm from normal to severe (p < 0.01). ICP showed a weaker correlation with papilledema grades (R^2^ = 0.339, p < 0.05), with significant increase (8 cm H_2_O, p < 0.01) only from normal to severe papilledema. RNFL correlated strongly with papilledema grade and moderately with ICP levels. RNFL thickness increased proportionally per papilledema grade.

## Introduction

Papilledema refers to swelling of the optic disc caused by elevated intracranial pressure (ICP). Papilledema and raised ICP without hydrocephalus or a mass lesion, along with normal cerebrospinal fluid (CSF) composition, constitute critical diagnostic criteria for idiopathic intracranial hypertension (IIH)^1,2^. The optic nerve is surrounded by three meningeal layers connected to the brain’s subarachnoid space. Such a connection allows increased ICP to affect the optic nerve directly^3,4^. Papilledema and lumbar puncture (LP) showing elevated CSF pressure are essential to prove IIH and exclude secondary causes^1,5^.

For diagnosis of IIH, measuring ICP via LP is a relatively direct and quick method, but it is invasive and unstable. ICP via LP can be affected by different factors such as body position, intra-abdominal pressure, patients’ collaboration, and even circadian rhythm^6,7^. In contrast, assessment of the papilla is non-invasive and relatively stable; however, inadequate for timely and precise monitoring of IIH. Frisén scale is a grading system to assess the severity of papilledema by evaluating the appearance of the optic disc and peripapillary retinal blood vessels^8,9^ It is widely used in clinical practice and research^10–13^. Frisén scale is ordinal with 0–5 grades, and has difficulties in discriminating small changes^14^. Further, the evaluation of papilla is most subjective and depends on clinical experience and fundus imaging quality, which limits its reproducibility, reliability, and sensitivity^15–17^. Therefore, relying solely on ICP level and papilledema grading alone cannot provide a precise measurement of the extent or progression of papilledema and may mislead clinical management.

Optical coherence tomography (OCT) is a technique that captures cross-sectional images of the retina and optic nerve head^18^. With its integrated analysis system, OCT can segment, calculate, and report on optic disc and macular structures automatically, providing both qualitative and quantitative measurements^19–21^. For monitoring ophthalmic diseases such as glaucoma, macular edema, OCT offers more accurate, reproducible, objective, and convenient quantitative measurements. Enhanced depth imaging OCT is particularly valuable in distinguishing optic disc drusen from papilledema^22,23^. In the last decade, OCT has been increasingly applied in the fields of neuroopthalmology and neuroinflammation to monitor the disease course and prognosis including IIH^24–28^.

One of the most well-supported theories regarding the pathogenesis of papilledema is axoplasmic stasis^3,29^. Raised ICP causes high pressure across the lamina cribrosa. Energy-dependent axoplasmic transport is subsequently arrested resulting in the accumulation of axoplasm in the lamina cribrosa leading to prelaminar intraaxon edema, which shows swelling of the optic nerve head, e.g. the papilla. The axons around the papilla originating from ganglion cells in the macula- can be quantified using OCT as peripapillary retinal nerve fiber layer (RNFL)^30^. Therefore, OCT theoretically measures papilledema through RNLF in a direct manner at a microscopic level with few micrometers’ resolution.

The study aims to evaluate the sensitivity and reliability of RNFL thickness in quantifying papilledema grade with correlation to ICP. To our knowledge, this is the first study to investigate the relationship of RNFL thickness, ICP and papilledema in patients with AIIH with papilledema and CIIH without papilledema but with previously diagnosed AIIH.

## Results

### Demographics and OCT measures in IIH and HC

A total of 104 patients diagnosed with idiopathic intracranial hypertension (IIH) and 80 healthy controls (HC) underwent OCT examinations (Table 1). The mean ages of the IIH and HC groups were 34 ± 12.2 and 35.5 ± 11.5 years, respectively (P > 0.05). Female participants predominated in both groups, comprising 87.5% of the IIH group and 82.5% of the HC group (p > 0.05) (Table 1). Among the 104 IIH patients, 65 presented with active IIH (AIIH) accompanied by papilledema, while 39 exhibited chronic IIH (CIIH) without papilledema but with a previous history of AIIH with papilledema. There were no statistically significant differences in age and sex between the two IIH groups compared to HC.

**Table 1:** Demographic characters of study groups.

Group	No	Age, mean ± SD	Sex, F/M (%)
IIH	104	34 ± 12.2	91/13 (87.5/12.5)
AIIH	65	32.6 ± 12.3	36/3 (92.3/7.7)
CIIH	39	36.1 ± 11.7	55/10 (84.6/15.4)
HC	80	35.5 ± 11.5	66/14 (82.5/17.5)

Mann–Whitney U test for age comparison and Chi-Square test for sex comparison between two groups. ANOVA test followed by post hoc analysis of Tamhane’s tests due to unequal variances of multiple groups. No significance was observed between the groups.

*IIH* idiopathic intracranial hypertension, *AIIH* active IIH, *CIIH* chronic IIH, *HC* health controls, *F/M* female/male.

The mean of RNFL thickness was significantly thicker in patients with AIIH with papilledema compared to those with CIIH without papilledema and HC in both right and left eyes (p < 0.01 for all comparisons) (Table 2). The mean of GCIPL thickness tended to be thinner in both AIIH and CIIH patients compared to HC in both right and left eyes. However, statistically significant thinning of GCIPL was observed only in the right eyes in CIIH (78.6 ± 8.6 µm, p < 0.05) but not in AIIH (79.6 ± 12.0 µm) when compared to HC (82.6 ± 5.2 µm).

**Table 2:** Optical coherence tomography (OCT) measures of study groups.

OCT measure	IIH (*n* = 104)	AIIH (n = 65)	CIIH (n = 39)	HC (*n* = 80)
RNFL (μm) mean ± SD				
Right	158.2 ± 107.4**	196.6 ± 120.2^##^	94.1 ± 14.3	95.5 ± 8.4
Left	153.0 ± 98.4**	187.9 ± 110.4^##^	94.9 ± 13.0	95.1 ± 8.8
Worst eye RNFL mean ± SD	169.6 ± 111.1**	212.9 ± 121.1^##^	97.5 ± 13.5	96.4 ± 8.6
Quadrant RNFL mean ± SD				
Temporal	96.4 ± 72.2	115.8 ± 86^##^	65.4 ± 14.9	64.7 ± 9.9
Superior	198.7 ± 140.5	252.3 ± 156.5^##^	112.9 ± 17.4	118.5 ± 14.1
Nasal	140.8 ± 98.3	179.3 ± 108.3^##^	79.23 ± 14.6	74.5 ± 14.5
Inferior	226 ± 143.2	286.3 ± 153.5^##^	129.5 ± 23.7	127.2 ± 16.6
GCIPL (μm) mean ± SD				
Right	79.3 ± 10.8	79.6 ± 12.0	78.6 ± 8.6^#^	82.6 ± 5.2
Left	79.9 ± 13.1	79.1 ± 15.7	80.2 ± 7.2	82.5 ± 5.2
Average	79.4 ± 11.3	79.3 ± 13.1	79.4 ± 7.6	81.4 ± 5.2

*IIH* idiopathic intracranial hypertension, *AIIH* active IIH, *CIIH* chronic IIH, *HC* health controls, *GCIPL* ganglion cell and inner plexiform layer, *RNFL* retinal nerve fiber layer.

Data are given as mean ± SD; **p < 0.01 according to Mann–Whitney U test between IIH and HC; #p < 0.05, ##p < 0.01 according to ANOVA test followed by post hoc analysis of multiple groups.

### Clinical and ophthalmic characteristics of AIIH and CIIH subgroups

All IIH patients underwent comprehensive neurological examinations, ophthalmological assessments, and neuroimaging. There were no significant differences observed in age, sex distribution, body mass index (BMI), and duration of symptoms between the AIIH and CIIH groups (Table 3). In both subgroups of IIH, female sex is dominant, which is in agreement with previous study^31^. Among the 65 patients with AIIH, 33.8% had mild, 35.4% had moderate, and 30.8% had severe papilledema. A total of 81 IIH patients, including 55 AIIH and 26 chronic CIIH, underwent ophthalmic examinations with OCT and fundus imaging and lumbar punctures either on the same day or within less than one week without prior therapy. The data from these patients were included in the correlation and regression analyses among RNFL thickness, ICP and the grade of papilledema.

**Table 3:** Clinical and ophthalmic characters of AIIH versus CIIH.

Clinical characteristics	AIIH (*n* = 65)	CIIH (*n* = 39)
BMI, kg/m^2^ mean ± SD (range)	32.9 ± 7.2 (20–55)	32.4 ± 5.8 (22–45)
Duration, months, mean ± SD (range)	11 ± 29 (0–190)	24 ± 46 (0–279)
ICP in cm CSF, mean ± SD (n, range)	36 ± 9 (n = 55, 20–60)	27 ± 8 (n = 26, 15–48)
Papilledema (n (%)		
Normal	0 (0)	39 (100)
Mild	22 (33.8)	
Moderate	23 (35.4)	
Severe	20 (30.8)	

*AIIH* active idiopathic intracranial hypertension, *BMI* body mass index, *CIIH* chronic IIH, *ICP* intracranial pressure.

### RNFL thickness in the worst eye of AIIH and CIIH subgroups compared to HC

The mean RNFL thickness of the worst eye in AIIH was significantly thicker (212.9 ± 121.1 µm) when compared to the worst eye RNFL in CIIH (97.5 ± 13.5 µm) and in HC (96.4 ± 8.6 µm) groups (p < 0.01 for all comparisons). Further analysis of quadrant-specific RNFL thickness in the most affected eyes showed that the mean RNFL thickness in all four quadrants (temporal, superior, nasal, and inferior) was significantly higher in AIIH than in CIIH and HC (p < 0.01 for all, Table 2). The distribution pattern of RNFL thickness across quadrants remained consistent in AIIH, CIIH as well as HC, following the order: temporal < nasal < superior < inferior.

### Correlation of RNFL thickness, ICP and papilledema grade

The correlation analysis was performed to explore the relationships of ICP, RNFL and the grade of papilledema. Patients with IIH were included for correlation analysis if LP was performed within one week without prior therapy. Fifty-five of 65 AIIH and 26 of 39 CIIH were included in correlation analysis.

**Fig. 1:**
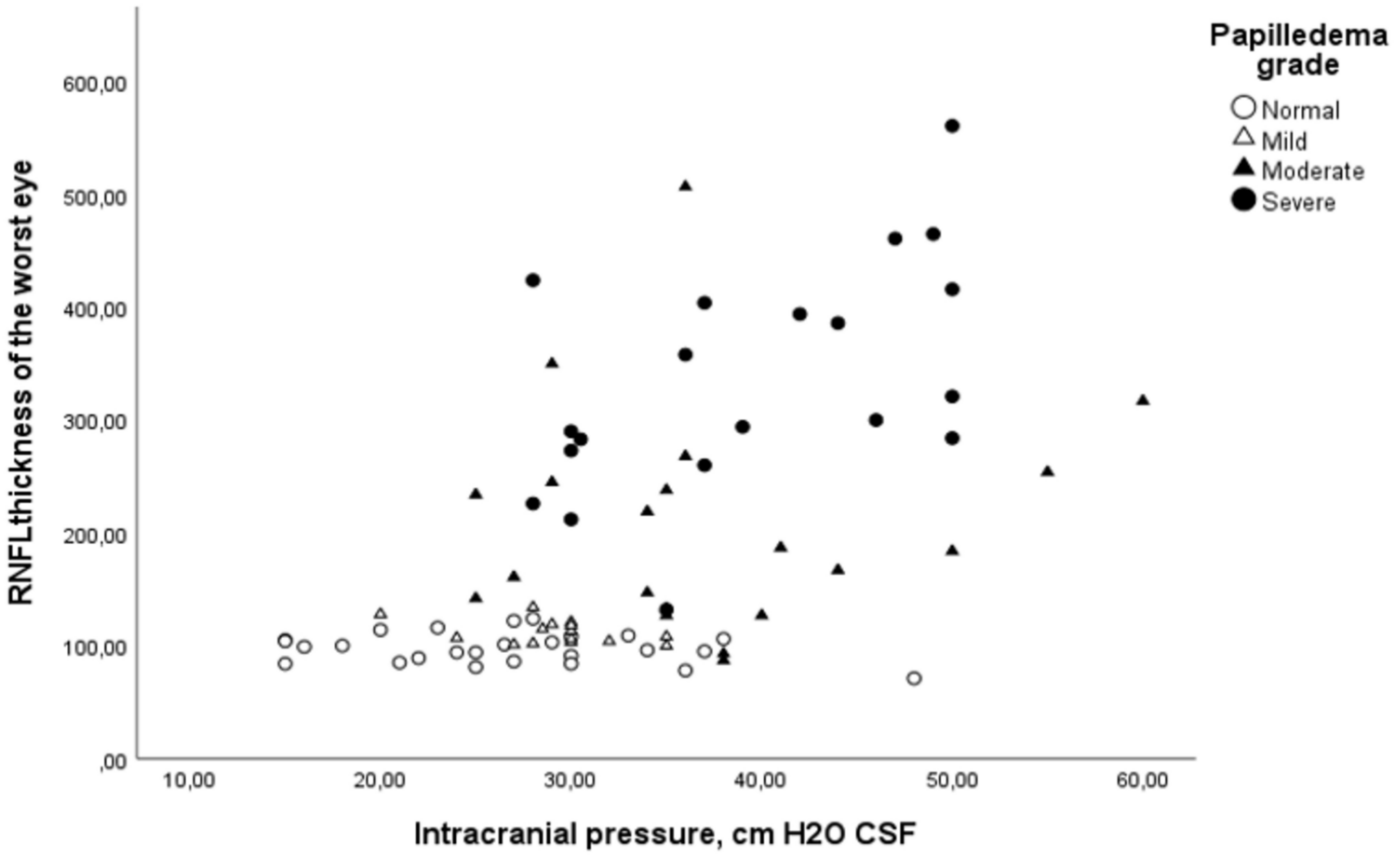
Correlation between retinal nerve fiber layer (RNFL) thickness (in micrometers) and intracranial pressure (ICP, in cm H2O CSF) across various papilledema grades in 80 patients with IIH. Coefficient is 0.78 between RNFL thickness and papilledema grade, 0.546 between RNFL and ICP, 0.569 between ICP and papilledema severity.

The results indicated a positive correlation between ICP and RNFL thickness (correlation coefficient = 0.546, p < 0.01) (Fig. 1), as well as between ICP and papilledema severity (correlation coefficient = 0.569, p < 0.01). A stronger positive correlation was observed between RNFL thickness and papilledema severity (coefficient 0.78, p < 0.01).

**Fig. 2:**
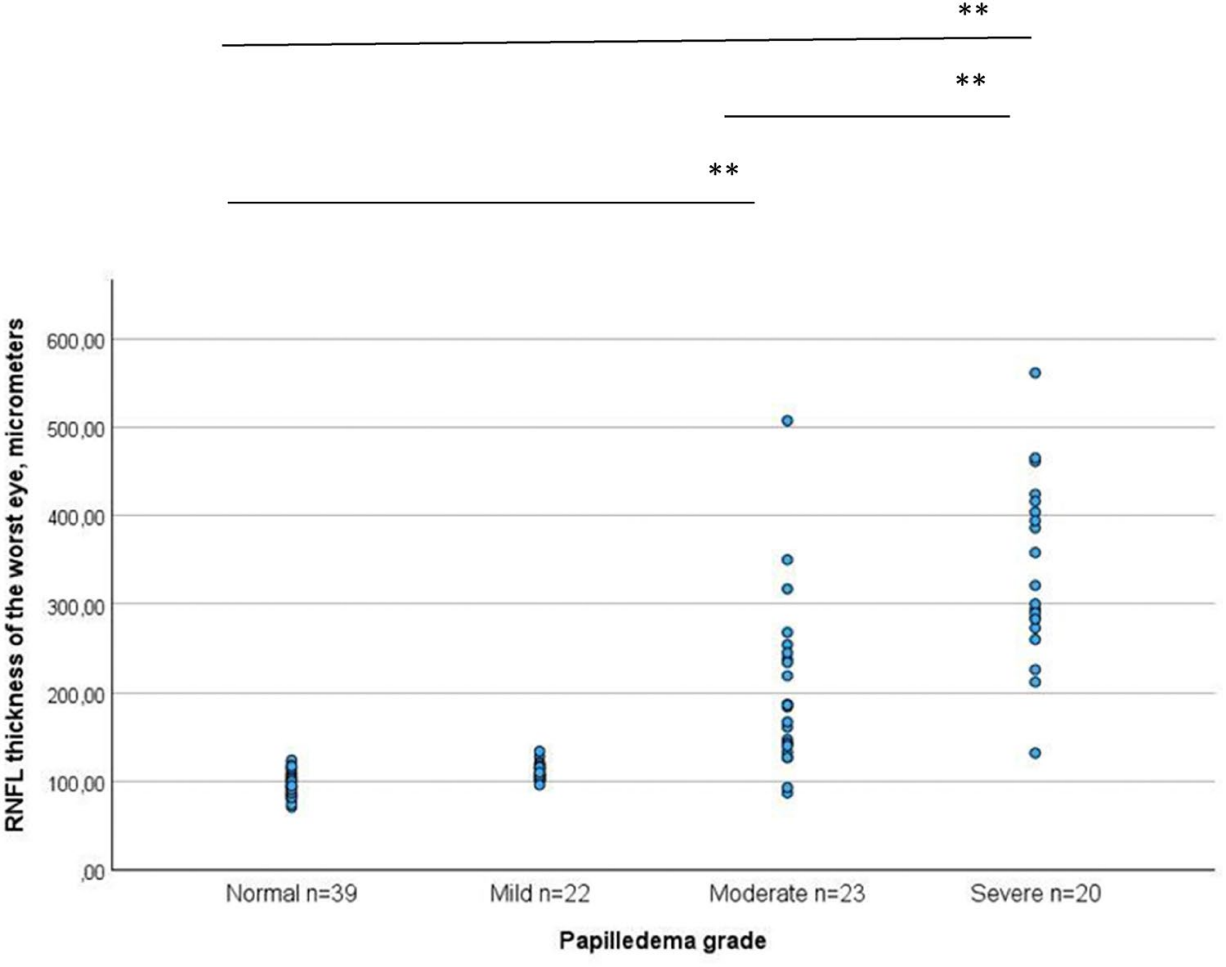
Retinal nerve fiber layer (RNFL) thickness in micrometers vs various papilledema grades. RNFL thickness is significantly higher in severe papilledema group compared to normal papilla, mild papilledema or moderate papilledema group. There is also a significant difference in RNFL thickness between the moderate and severe papilledema groups. **p < 0.01 according to ANOVA test.

To further validate the results, ANOVA with post hoc corrections was conducted. When comparing RNFL thickness across different grades of papilledema, the most substantial RNFL thickness was observed in the severe papilledema group (337 ± 102 µm). This measurement was significantly different from those in the normal papilla (98 ± 14 µm), mild papilledema (111 ± 9.4 µm), and moderate papilledema (202 ± 95 µm) groups (p < 0.01 for all comparisons). RNFL thickness in the moderate papilledema group also differed significantly from normal, mild and severe papilledema groups (p < 0.01 for all comparisons). There was no statistically significant difference in RNFL thickness between the normal papilla and mild papilledema groups (Fig. 2).

**Fig. 3:**
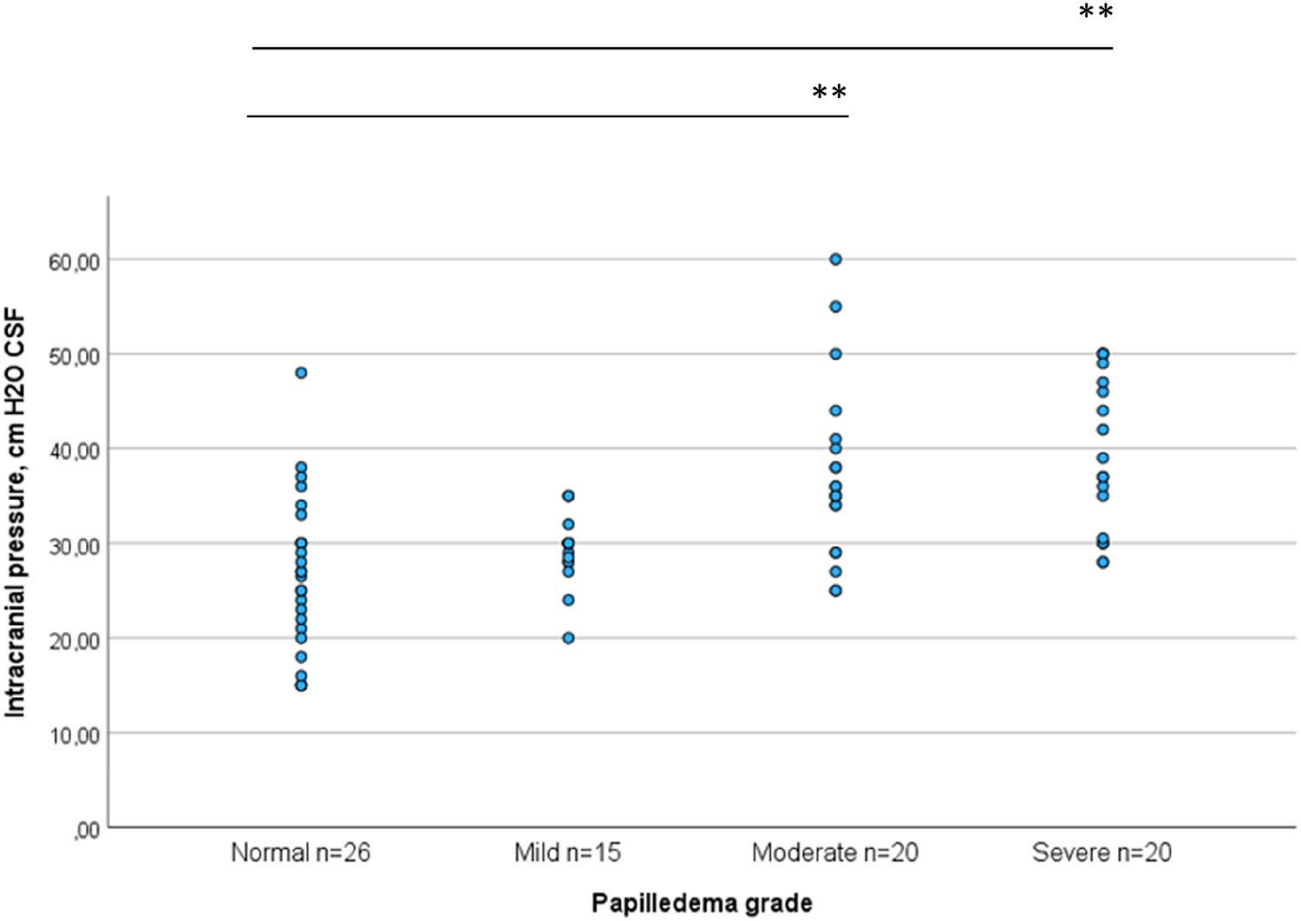
Intracranial pressure (ICP) measured via lumbar puncture in cm H_2_O vs various papilledema grades. ICP was significantly higher in moderate and severe papilledema groups compared to normal papilla and mild papilledema groups. No significant difference in ICP between normal papilla and mild papilledema groups, nor between moderate and severe papilledema groups. **p < 0.01 according to ANOVA test.

ICP was compared across different grades of papilledema. Significant differences in ICP were observed between the normal papilla group (26.3 ± 8.1 cm H_2_O) and both the moderate (37.3 ± 9.3 cm H_2_O) and severe (39.4 ± 8.3 cm H_2_O) papilledema groups. Additionally, ICP was significantly lower in the mild papilledema group (29.1 ± 3.8 cm H_2_O) when compared to the moderate and severe papilledema groups (p < 0.01 for both). However, no significant difference was found between the normal papilla and mild papilledema groups, and no difference was observed between the moderate and severe papilledema groups (Fig. 3).

### Strong association of RNFL thickness and papilledema grades

Linear regression analysis revealed a significant association between papilledema severity with RNFL thickness (adjusted R^2^ = 0.656, p < 0.01). Specifically, an increase in papilledema grade from normal to severe corresponds to an increase in RNFL thickness by 214 µm (p < 0.01); from normal to moderate, an increase in RNFL thickness by 91 µm (p < 0.01); and normal to mild, an increase in RNFL thickness by 9 µm (p = 0.68).

Additionally, linear regression analysis showed a significant association between papilledema severity and ICP thickness (adjusted R^2^ = 0.339, p < 0.05). An increase in papilledema grade from normal to severe is associated with an increase in ICP by 8 cm H_2_O (p =  < 0.01); from normal to moderate grade, an increase in ICP by 7 cm H_2_O (p = 0.056); from normal to mild grade, an increase in ICP by 2 cm H_2_O (p = 0.4).

## Discussion

To our knowledge, this is the first study to investigate the relationship between RNFL thickness, ICP level, and papilledema grade in patients with AIIH with papilledema and CIIH without papilledema, but with previously diagnosed AIIH^32–34^. Our findings indicate that RNFL thickness is significantly higher in AIIH compared to CIIH and HC. Furthermore, RNFL thickness shows a stronger correlation with papilledema grade than ICP level of IIH. As the severity of papilledema increases, there is a corresponding thickening of the RNFL, whereas the relationship between ICP and papilledema grade is not as robust. The RNFL thickness increases more proportionally per papilledema grade than ICP levels, suggesting that RNFL thickness is a more precise and reliable measurement for assessing IIH severity, progression and regression.

OCT has transformed ophthalmology over the last two decades^19,21,24^. OCT can make a similar impact in neurology by monitoring the optic nerve disc and its neurons in the eye fundus through measurements of RNFL and GCIPL thickness^30,35,36^. In this study, we observed a significant increase in RNFL thickness in AIIH with papilledema, while RNFL remained normal in CIIH group without papilledema. There was no significant difference in RNFL thickness between the CIIH and HC. Further quadrant-specific RNFL analysis showed similar results: RNFL thickness in all four quadrants was significantly greater in AIIH with papilledema than in CIIH without papilledema. However, the pattern of quadrant thickening remained consistent between the two groups, following the previously established order: temporal < nasal < superior < inferior^34,37^, and showed a similar correlation with ICP levels in cases of papilledema^34^.

We found that mild papilledema exhibited thicker RNFL than normal papilla, though the difference was not statistically significant, consistent with findings from previous studies^9,38–40^. ICP levels were only slightly above the normal reference range, highlighting the challenge of distinguishing these conditions^41–43^ based solely on these parameters. ICP measurement and mild papilledema alone or combined are not reliable for making a definitive diagnosis. A thorough ophthalmological examination is essential to rule out conditions such as drusen, glaucoma, extreme refractive errors, or optic neuritis. Longitudinal follow-up using OCT is highly valuable for monitoring dynamic changes in RNFL, especially during treatment. Furthermore, assessing longitudinal changes in RNFL ideally including baseline measurements alongside GCIPL evaluation is of great help. Unlike in glaucoma and optic neuritis, GCIPL is often preserved in AIIH and is rarely affected by ICP levels, even over the long term.

In our study, GCIPL thickness was marginally reduced in the right eye of the CIIH group compared to HC; however, the average GCIPL thickness did not differ significantly between the groups. Long-term elevated ICP can affect the visual pathway, leading to thinning of both GCIPL and RNFL through an unknown mechanism^3,4^. However, this is rare if treatment is initiated in time. Future research is needed to clarify the relationship between GCIPL thinning, papilledema grades, ICP levels, and visual function. GCIPL may serve as a complementary biomarker for evaluating disease progression and treatment outcomes.

Anatomically, the RNFL consists of optic nerve axons originating from the macular GCIPL and communicates with the brain’s CSF space at the lamina cribrosa^44^. Changes in ICP should therefore be promptly reflected in the RNFL thickness with minimal delay between ICP transmissions and corresponding RNFL thickness alterations. This should underscore the superiority of measuring RNFL thickness over relying on observable changes in fundus images for monitoring IIH course. However, it is important to consider anatomical variations in the optic nerve sheath, optic canal, craniospinal compliance distribution, and intraocular pressure^3,29,45,46^, especially when patients with CIIH present with a normal optic disc or/and AIIH with mild papilledema. Severe papilledema and long-term elevated ICP can lead to irreversible visual deterioration^10,21,47^. Even minor changes in RNFL thickness, measured in just a few micrometers, can indicate subtle ICP changes. This can guide physicians to take action, even if the appearance of the optic disc (papilla) remains unchanged or appears normal.

At this stage, GCIPL results should be interpreted with caution, as GCIPL thickness in AIIH can be affected by optic disc edema, potentially leading to skewed values. However, thinner GCIPL in IIH than in HC may indicate that elevated ICP or increased CSF volume may affect the visual pathway in these patients^30,48^. To further investigate this, a prospective longitudinal study examining the relationship between visual function and GCIPL thickness is warranted.

Although ICP measurement via LP is essential for diagnostic and occasionally a therapeutic tool^41,43^, it is invasive and has several drawbacks, including the possibility of a rebound effect^6,7,49^. Our results showed a significant correlation between ICP levels, papilledema grades and RNFL thickness (Fig. 1), but there was considerable variation of ICP within each papilledema grade group (Fig. 3). ICP was significantly lower in the normal papilla and mild papilledema groups compared to the moderate or severe papilledema groups. However, there was no significant difference in ICP between the normal papilla and mild papilledema groups, nor between the moderate and severe papilledema groups. CIIH patients often had elevated ICP without papilledema^40^, and patients with chronic headaches, including migraines, frequently exhibited elevated ICP^42^. These findings, along with our current data, suggest that ICP via LP alone is not reliable to distinguish AIIH from CIIH, nor to determine papilledema severity.

Papilledema is primarily a mechanical phenomenon caused by the stasis of axoplasmic flow in the optic nerve fibers, resulting from elevated ICP caused by the accumulation of CSF around the myelin sheath of the optic nerve^3,50^. It often takes 1–5 days of persistently elevated ICP for papilledema to appear on ophthalmoscopy^8^, and the resolution of this edema can take several months. Therefore, the assessment of papilledema grade lacks precision and sensitivity and does not provide real-time information. In addition, unlike RNFL measurements, papilledema grade does not provide objective measurement of papilla atrophy and GCIPL information.

In conclusion, our study demonstrates that RNFL measurement is a rapid, precise, reliable, real-time, and objective tool for monitoring the course of IIH, surpassing fundus imaging and ICP measurement via LP. RNFL should be the primary method for follow-up and evaluating therapeutic response in IIH patients, while the combination of LP with ICP measurement along with fundus imaging including OCT remains a critical component of the diagnostic criteria.

A limitation of our study is the time lag between ICP measuremen via LP and the data collection for OCT examination and fundus imaging. Patients were routinely referred from ophthalmologists to neurologists for LP, which could lead to delays and may affect accuracy of the results. Another limitation is the grading papilledema, we followed partially the Frisén grade 0–5, with simplification using “normal = 0”, mild = 1, moderate = 2 and 3, severe = 4 and 5. Further, in severe papilledema, RNFL values can be inaccurate with large variations, and we performed several times examinations with OCT in these patients. Finally, not all healthy controls were examined by an ophthalmologist. Family history and medical history played a dominant role in the selection process, which may have introduced additional bias.

## Methods

### Study population

This cross-sectional study enrolled 104 consecutive patients with active or chronic IIH at the Neurological Division of Linköping University Hospital, Sweden, from May 2014 to May 2024. Eighty age- and sex-matched control subjects were included during the same period. The study was approved by Linköping University Review Board (2020-01532). Written signed informed consent forms were obtained from all participants or legal guardians if the participants were younger than 18 years old. All methods were performed in accordance with the relevant guidelines and regulations.

The diagnosis of IIH was made according to criteria established by Friedman et al.^1^ which include 1) papilledema, 2) normal neurological examination except for cranial nerve VI abnormalities, 3) neuroimaging showing normal brain parenchyma without hydrocephalus, mass or structural lesions, 4) normal cerebrospinal fluid (CSF) composition, 5) CSF opening pressure ≥ 25 cm H_2_O for adults and ≥ 28 cm H_2_0 for children. Among the patients, 65 had active or acute IIH (AIIH) with papilledema and 39 had previously confirmed AIIH but were in remission at chronic phase (CIIH) without papilledema at the time of recruitment.

Healthy controls (HC) were recruited from our staff, students and patients with primary headache, dizziness, and hyperesthesia. HC did not have hypertension, diabetes mellitus, optic neuritis, glaucoma or macular disorders. All HC were examined with OCT and fundus images.

### Diagnosis and grading of papilledema

The fundus images were obtained from both eyes of all the patients with IIH using either digital fundus camera or ophthalmoscopy. All the fundus images from IIH patients were evaluated by ophthalmologist and neurologist. Grading of papilledema was essentially based on the modified Frisén scale^8,9^ with certain clinical fits and simplification. The key features for grading papilledema include 1) grade 0 indicating normal findings of the papilla; 2) mild papilledema indicating papilledema with C-shaped halo and normal temporal disc margin; 3) moderate grade indicating circumferential halo with or without obscuration of major blood vessels leaving the disc; 4) severe grade indicating complete disc halo with major or total blood vessels obscuration.

### Optical coherence tomography

OCT images (Cirrus 4000, Carl Zeiss Meditec, CA, USA) were obtained in all subjects within two days after fundus assessment. The peripapillary retinal nerve fiber layer (RNFL) thickness was measured using the protocol of Optic Disc 200 × 200 over 3.4-mm diameter circle centered on the optic disc. The macular ganglion cell-inner plexiform layer (GCIPL) thickness was measured using the protocol of macular cube 512 A-scan × 218 B scan over a 6 × 6 mm^2^ area centered on the fovea.

The examination was performed without mydriasis. The optic disc and macula margin were bounded automatically by software without manual modification. Scans with signal strength ≥ 7 and no missing parts within the measurement circle were accepted^39,40^.

### CSF opening pressure is used to indicate intracranial pressure (ICP)

Lumbar puncture (LP) was performed on the patients within five days after OCT and fundus examination or before the treatment. The patients were in the left lateral decubital position. A 22-gauge atraumatic spinal needle was used. Once cerebrospinal fluid (CSF) was obtained, a spinal fluid manometer (Optidynamic, Mediplast, Italy) was connected to measure CSF opening pressure in cm CSF. In order to avoid artificially elevated CSF pressure, the patients were relaxed, with legs extended and with the neck in a neutral position. After waiting for a few min, CSF opening pressure was recorded.

CSF opening pressure measured via lumbar puncture was used to indicate intracranial pressure (ICP) at the moment.

### Statistical analysis

Statistical analyses were conducted using IBM SPSS Statistics, version 29. Depending on the data distribution, continuous variables were compared between groups using either an independent t-test or the Mann–Whitney U test, while categorical variables were compared using the Chi-Square test or Fisher’s exact test. Correlation analysis and ANOVA with post hoc tests were employed to explore the relationships and directions of the variables ICP, RNFL, and papilledema grades. Linear regression analysis was performed twice: first with ICP as the dependent variable with RNFL and papilledema severity as independent variables, and subsequently with RNFL as the dependent variable with ICP and papilledema severity as independent variables. Statistical significance was defined as a two-tailed p-value of ≤ 0.05.

## Data Availability

The datasets used and/or analyzed during the current study available from the corresponding author on reasonable request.

## Abbreviations

BMI: Body mass index
HC: Healthy controls
ICP: Intracranial pressure
IIH: Idiopathic intracranial hypertension
GCIPL: Ganglion cell-inner plexiform layer
LP: Lumbar puncture
OCT: Optical coherence tomography
RNFL: Retinal nerve fiber layer
